# The effects of plant and animal-derived aminoacid and humic acid-based biostimulants on yield and quality in the wine grape

**DOI:** 10.3389/fpls.2025.1659765

**Published:** 2025-09-19

**Authors:** Filiz Hallaç Türk, Aydın Kurt

**Affiliations:** Department of Horticulture, Faculty of Agriculture, Isparta University of Applied Sciences, Isparta, Türkiye

**Keywords:** grape, plant-derived amino acids, animal-derived amino acids, grape quality, grape yield

## Abstract

In recent years, biostimulants, which are of great importance in sustainable viticulture, are generally applied to plants at low doses via foliar application to regulate or enhance plant physiological processes, improve product vitality, yield, and quality, and overcome stress conditions. This study was conducted in Antalya, Turkey, in 2019 and 2020, to determine the effects of different biostimulant applications on yield and quality parameters in the Öküzgözü grape variety. The experiment was set up in a randomized block design with three replications. The treatments encompassed humic acid, plant-derived amino acids, animal-derived amino acids, and their combinations (HU+PAA and HU+AAA), with a control group receiving tap water. It is evident from the analysis of two-year averages that biostimulant applications have a considerable impact on the morphological characteristics of berries, including size, as well as on cluster characteristics and yield per cluster. The biochemical quality criteria also demonstrate a clear response to biostimulant application. The highest yield (13.20 kg·plant^-1^), cluster weight (505.5 g), and 100-berry weight (608.4 g) were obtained with the HU+PAA combination. The application of HU+AAA resulted in a significant increase in anthocyanin content (1463 mg·100 g ^-1^FW), while the application of plant amino acids led to a notable increase in L-ascorbic acid content (8.60 mg·100 g ^-1^ FW). Statistically significant differences were not observed between the treatments in terms of phenolic compound and flavanol contents. The creation of a heat map was informed by the data that was obtained, and principal component analysis (PCA) was subsequently performed. The findings indicated that biostimulant applications, particularly when used in combination, are efficacious in enhancing both yield and quality in the Öküzgözü grape variety.

## Introduction

Turkey is a country that has pioneered viticulture worldwide. It is extremely rich in grape genetic resources and has suitable ecological conditions (Çelik et al., 1998). Grape production is a strategic agricultural activity that contributes to the national economy and to healthy nutrition. However, increasing biotic and abiotic stress factors, the effects of climate change, soil organic matter depletion, and intensive chemical fertilizer use have negatively impacted vineyard yield and quality in recent years (Jones et al., 2005; Taban et al., 2013; Sabır et al., 2021).

There is, therefore, an increasing need for environmentally friendly practices that promote plant growth and enhance product quality to ensure sustainable viticulture. In this context, biostimulants have become important agricultural materials. These are compounds containing organic substances or microorganisms that enhance plants’ tolerance to stress and improve growth and quality by stimulating physiological activities (Du Jardin, 2015; Xu and Geelen, 2018). In particular, products containing humic and amino acids have positive effects on photosynthesis, water uptake, root development, and nutrient uptake in viticulture (Canellas et al., 2011; Colla et al., 2015; Rouphael et al., 2017).

In general, nine categories of substances that act as biostimulants have been defined: (1) humic substances; (2) complex organic materials (obtained from agro-industrial and urban waste, sewage sludge extracts, compost, and manure); (3) beneficial chemical elements (Al, Co, Na, Se, and Si); (4) inorganic salts, including phosphite; (5) algae extracts (brown, red, and green macroalgae); (6) derivatives of chitin and chitosan; (7) antiperspirants (kaolin and polyacrylamide); (8) free amino acids and N-containing substances (peptides, polyamines, and betaines); and (9) plant growth promoting rhizobacteria (PGPR), arbuscular mycorrhizal fungi (AMF), and *Trichoderma* spp (Rouphael and Colla, 2020; Monteiro et al., 2022).

Biostimulants can generally increase vitality, promote vegetative growth, improve the absorption and distribution of nutrients within the plant, increase antioxidant capacity, and improve tolerance to biotic and abiotic stresses, thereby increasing plant yield and fruit quality (García-Sánchez et al., 2022). Additionally, it has been reported that the external application of elicitors promotes the activation of enzymes involved in the synthesis of phenolic compounds and thus plays an important role in plant-pathogen interactions (Gutiérrez-Gamboa et al., 2019; Monteiro et al., 2022).

Viticulture is facing increasing abiotic and biotic stress factors due to climate change. However, the main goal of all winegrowers is to maintain the quality of grapes and wine. At the same time, it is necessary to reduce chemical use and make viticulture more sustainable and environmentally friendly. For this reason, the use of biostimulants in vineyards is increasing day by day in the world and in Turkey to overcome the problems encountered in grape cultivation, which is one of the most valuable fruit types globally (Bernardo et al., 2018). Du Jardin (2015) noted that table and wine grapes are among the primary products in Europe where biostimulants are applied. Monteiro et al. (2022) identified the most commonly used biostimulants in vineyards as kaolin for abiotic stress alleviation, stinging nettle (*Urtica dioica*) and Japanese knotweed (*Fallopia japonica*), abiotic and biotic stress alleviators such as seaweed extracts, chitosan, yeast extracts, plant regulators, and elicitors such as methyl jasmonate, abscisic acid, salicylic acid, and glycine betaine. Additionally, Gutiérrez-Gamboa et al. (2019) have noted that formulations based on urea, amino acids, and commercial nitrogen fertilisers are the most commonly used biostimulants in vineyards to improve grape and wine quality. They also mention that elicitors can trigger defence reactions in vines by inducing the synthesis of secondary metabolites, primarily phenolic and volatile compounds.

Among the biostimulants applied to vines, products containing humic acid and amino acids have positive effects on photosynthesis, water uptake, root development and nutrient uptake in viticulture (Canellas et al., 2011; Colla et al., 2015; Rouphael et al., 2017). Humic acids not only improve the physical and microbiological structure of the soil, but also stimulate plant metabolism when applied to the leaves, thereby increasing yield and quality (Ferrara and Brunetti, 2010; Popescu and Popescu, 2018). The effects of humic acid application in plants generally involve the activation of plasma membrane H+-ATPase and changes in primary and secondary metabolism, which typically result in increased root growth, nutrient uptake, photosynthesis rate, and reduced stress associated with salinity, drought, or metal toxicity (Dobbss et al., 2018; Cataldo et al., 2022; Jindo et al., 2022). Amino acids, when applied to plants in the form of protein hydrolysate, have effects on chlorophyll biosynthesis, enzyme activation, and stress adaptation (Bulgari et al., 2019; Boselli et al., 2019). Protein hydrolysates are important plant biostimulants based on mixtures of peptides and amino acids produced by the enzymatic and/or chemical hydrolysis of proteins from mainly animal or plant-based raw materials (Colla et al., 2015). The fact that amino acids from plant and animal sources exhibit different effects highlights that the source and dose are important variables (Cerdán et al., 2009; Botta, 2013). To use these diverse biostimulants effectively in vines, it is important to determine the different mechanisms of action of their bioactive compounds. However, the mechanisms triggered by these biostimulants in plants are still not fully understood. Since the responses to the application of the same biostimulants may vary depending on the conditions the plant is exposed to, it is recommended to study different grape varieties and terroirs (Monteiro et al., 2022).

This study determined the effects of humic acid, plant- and animal-derived amino acids, and combinations of these substances on vine yield and grape quality in the “Öküzgözü” grape variety grown in Antalya, Turkey. The study revealed the feasibility of using environmentally friendly biostimulants as an alternative to traditional practices in viticulture and their contribution to the production performance of the Öküzgözü grape.

## Materials and methods

### Experimental site

The field experiments were carried out in a wine grape variety ‘Öküzgözü’ (*Vitis Vinifera* L.) vineyard located in Elmalı, Antalya (Turkiye) (36°37’08.8”N 29°57’24.1”E and altitude at 1100 m above sea level) over two growing seasons (2019 and 2020). The vines were 13-year-old ‘Öküzgözü’ vines grown on their own roots in a commercial vineyard. The vines had been pruned as Guyot double with mix of canes and spurs. The vines were planted at 3.0 m between rows and 1.5 m within rows (3472 vines per hectare) with a North–South row orientation.

**Figure 1:**
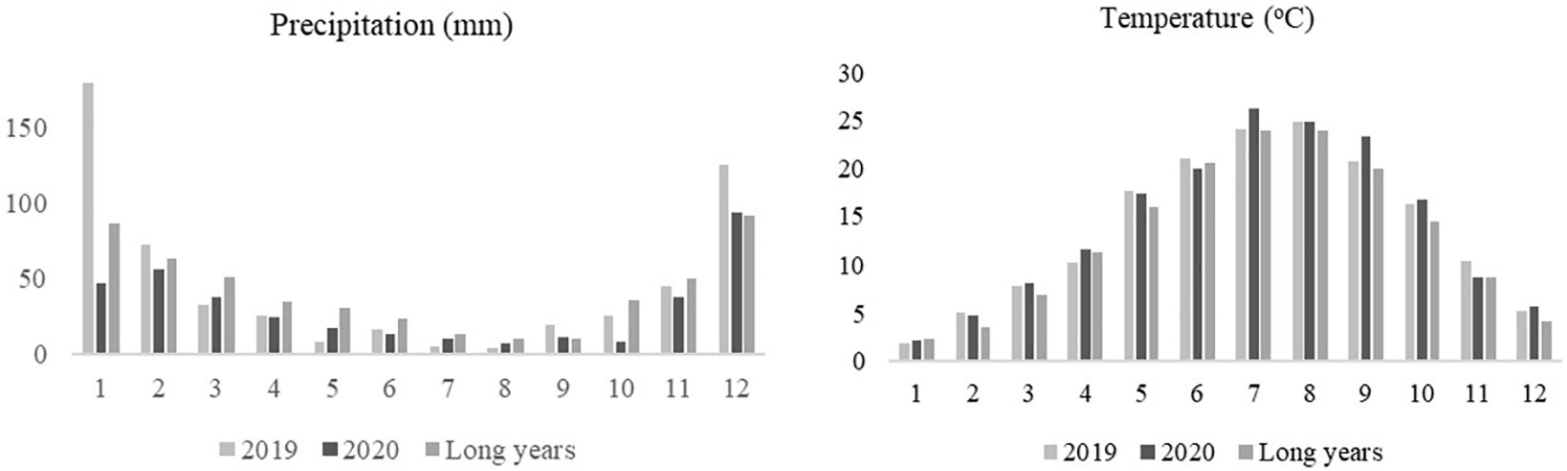
Monthly rainfall and temperature data for the experimental area.

The research area has a soil structure characterized by a slightly alkaline pH (7.5), a clay-loam texture, a high lime content (16.3%), a low organic matter content (1.9%), and sufficient levels of macronutrients and micronutrients. During the 2019-2020 season, climate data for the region was obtained from the General Directorate of Meteorology. The information included monthly temperature and rainfall (**Figure 1**).

### Characterization of the biostimulants used

In this study, the commercial preparations Vulcano (plant-derived amino acids), Colostrum (animal-derived amino acids), and BlackJak (humic acid) were used as biostimulants.

Colostrum is a nanotechnological foliar fertilizer containing micronized particles and consisting of 44% organic matter, 24% organic carbon, 6.8% organic nitrogen, and 14% free amino acids. Vulcano consists of 40% organic matter, 20% organic carbon, 2% organic nitrogen, 4.5% potassium oxide, and 10% free amino acids. BlackJak, a liquid humic acid, contains 15% organic matter, 15% humic and fulvic acids, and 0.003% potassium oxide.

### Plant material and experimental design

The research was conducted on the “Öküzgözü” wine grape variety grown on its own roots. Named for the size of its berries (6 g), the “Öküzgözü” grape variety is elliptical in shape, greyish-black in colour, seeded, and has medium-thick skin. The clusters are winged conical in shape, full, and large (450-550 g). It is mainly cultivated in the Elazığ, Gaziantep, and Malatya regions. It is a late-ripening variety that matures in late September to mid-October. It is consumed both as wine and table grapes. This moderately productive variety is suitable for mixed pruning of canes ans spur. Its world-renowned wine has a distinctive aroma. It is full-bodied, robust, and of high quality. The wine has high acidity and low alcohol content (Çelik, 2006).

The field trial was established for two consecutive growing seasons: 2018-2019 and 2019-2020. In a factorial experimental design, three different biostimulants, their combinations and control were used as treatments. Biostimulant applications were carried out in six groups (**Table 1**).

**Table 1:** Biyostimulants treatments.

Treatments	Biostimulants	Application doses
C	Control	Only tap water application
HU	Humic acid	0.05 ml·L^-1^ BlackJak
PAA	Plant-derived amino acid	0.2 ml·L^-1^ Vulcano
AAA	Animal-derived amino acid	0.2 ml·L^-1^ Colostrum
HU+PAA	Plant-derived amino acid+ Humic acid	0.05 ml·L^-1^ BlackJak + 0.2 ml·L^-1^ Vulcano
HU+AAA	Animal-derived amino acid+ Humic acid	0.5 ml·L^-1^ BlackJak + 0.2 ml·L^-1^ Colostrum

Treatment plots were separated from each other by an untreated row. In order to minimize possible contamination effects from nearby treatments, data was collected only from the inner rows of each plot. During each biostimulants application the foliage of each plant was fully wetted. The control plants were manually sprayed with tap water alone. Applications were initiated when the shoots reached a height of 15–20 cm (9.05.2019 and 06.05.2020), four times carried out at 15-day intervals (including reblooming and fruit settings). The biostimulants were applied to the leaves via a spraying method. Harvesting took place at the beginning of October each year.

### Measured parameters

#### Determination of the effect of humic acid, plant and animal derived amino acid on grape yield and quality

In order to measure yield per vine (kg), clusters collected from each vine per application were immediately weighted using a digital balance and divided by vine number. Total cluster weight per application was weighted using a digital balance and divided by cluster number to record the average weight of clusters (g). The width and length of five clusters from each vine were measured using a digital caliper, and the average was calculated. Three clusters from each vine per application were selected in order to evaluate average berry weight. The width and length of 20 berries per cluster were measured in millimeters using a digital caliper. 100 grape berries collected from the upper, middle, and lower sections of the clusters were weighed using a digital balance and divided by berry number to calculate average berry weight (g).

Fruit juice samples were obtained randomly from 10 berries in a different part of clusters for the quality analyses. Total soluble solid (TSS) of berry juice was determined by using a digital refractometer at 20 C and the results were expressed as Brix. Titratable acidity (TA) was determined by titration with 0.1 N NaOH up to pH 8.1. by using 10 mL of diluted juice and the results were expressed as g tartaric acid L^−1^ grape juice. The grape juice pH was subsequently measured using a pH meter. The maturity index is obtained by dividing Brix value by the titratable acidity. The berry color was analyzed using a colorimeter CR-10 (Minolta^®^, Tokyo, Japan) to obtain the following variables from the equatorial portion of berries (n = 2 per berry): *L* (lightness), C* (chroma), and *h* (hue) (Koyama et al., 2014). Lightness values range from 0 (black) to 100 (white). Chroma indicates the purity or intensity of color; the distance from gray (achromatic) toward a pure chromatic color is calculated from the a* and b* values of the CIELab scale system, which starts from zero for a completely neutral color, and does not have an arbitrary end, but intensity increases with magnitude. Hue refers to the color wheel and is measured in angles; green, yellow, and red correspond to 180, 90, and 0, respectively (McGuire, 1992; Lancaster et al., 1997).

#### Determination of grape antioxidant components

The total phenolic compound content was determined using the Folin-Ciocâlteu colorimetric method as described by Singleton and Rossi (Singleton and Rossi, 1965). The reduction of the Folin– Ciocâlteu reagent by phenolic compounds under alkaline conditions, which resulted in the development of a blue color, was measured 765 nm (UV–vis model 1601, Shimadzu, Kyoto, Japan). The amount of total phenolic was calculated with the use of a calibration curve made from gallic acid standard and expressed as mg gallic acid g^−1^ FW equivalents. The total flavanol content was determined by the DMAC (dimethylamino cinnamaldehyde) method (Arnous et al., 2001). Spectrophotometric readings were obtained at a wavelength of 640 nm. The total flavanol content was determined as catechin equivalents in milligrams per gram (mg CE·g^-1^) of FW equivalents. The total anthocyanin content was determined using the pH differential method (Wrolstad, 1976). For this purpose, aliquots of the extracts were adjusted to pH 1.0 and 4.5 with buffers. The absorbance of each solution was measured at a wavelength of 520 and 700nm. L-Ascorbic acid content was determined spectrophotometrically at 520 nm by using a standard curve according to the procedure described by Pearson and Churchill (1970). Each determination was carried out in triplicate.

### Statistical analysis

The study was conducted using a randomised block design with, three replications and four vines per replication (totalling 72 vines) in sepaerate vine rows. The two-year data obtained from the research were subjected to the Levene homogeneity test; combined variance analyses were performed on the data determined to be homogeneous. The obtained data were analyzed using Minitab 17 statistical software, with one-way analysis of variance (ANOVA) employed to test for significance. When differences were found to be significant, means were compared using the least significant difference (LSD) test, with a significance level of P<0.05 and P<0.01. Principal component analysis (PCA) and heatmap analysis were performed using the RStudio V4.3.1 program (RStudio Team, 2023; R Core Team, 2023, Vienna, Austria).

## Results

### Yield parameters

**Figure 2:**
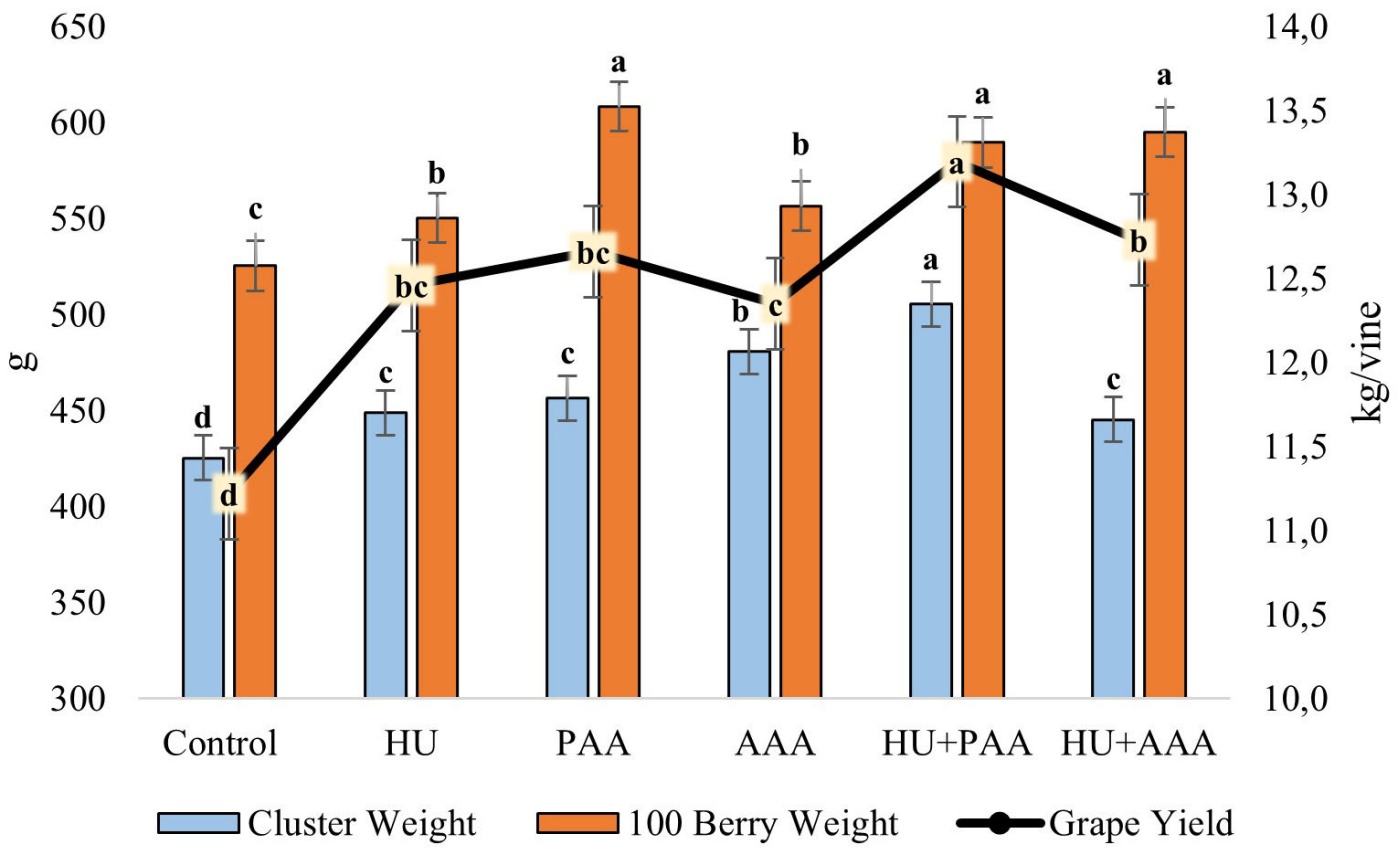
Cluster weight (g), 100 berry weight (g) and yield (kg/vine) values are presented as the mean ± standard error bars. Different letters indicate significant differences (p ≤ 0.05) among different biostimulant applications.

**Figure 3:**
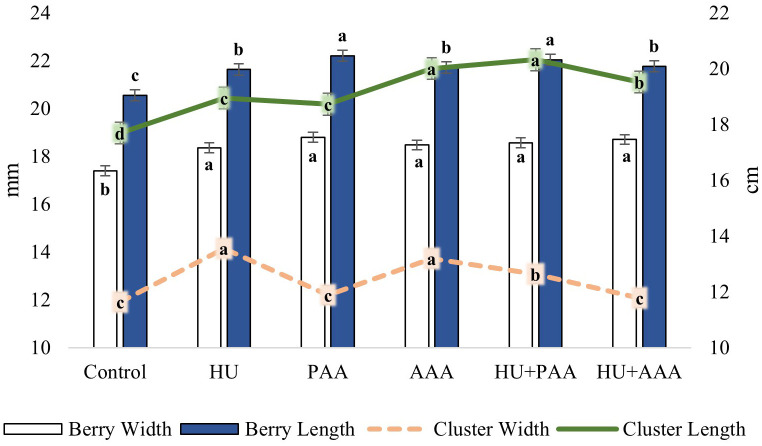
Berry width and length (mm), cluster width and length (cm) values are presented as the mean ± standard error bars. Different letters indicate significant differences (p ≤ 0.05) among different biostimulant applications.

The response of biostimulantson yield and quality shown in **Figures 2** and **3**. Different biostimulant applications had a statistically significant effect on vine yield, cluster weight, and 100-berry weight. The highest vine yield (13.20 kg) was achieved with the HU+PAA, while the lowest yield (11.22 kg) was achieved with the control application (**Figure 2**). The lowest cluster weight was 425.3 g in the control, while the highest was 505.5 g in the HU+PAA. The lowest 100-berry weight value (525.4 g) was found in the control. PAA (608.4 g), HU+AAA (595.2 g) and HU+PAA (589.7 g) had high values and were statistically grouped together. According to the results of the variance analysis, statistically significant differences were determined between biostimulant applications in terms of berry width, berry length, cluster width and cluster length values. The lowest values for all four characteristics were obtained in the control (**Figure 3**). In terms of berry width, all biostimulant applications were in the same statistical group, with values ranging from 18.35 to 18.80 mm. The highest berry length values were obtained in the PAA and HU+PAA, with 22.21 and 22.04 mm, respectively. The highest cluster width values were 13.56 cm and 13.19 cm in the HU and AAA, while the highest cluster length values were 20.00 and 20.32 cm in the AAA and HU+PAA.

### Quality parameters

**Figure 4:**
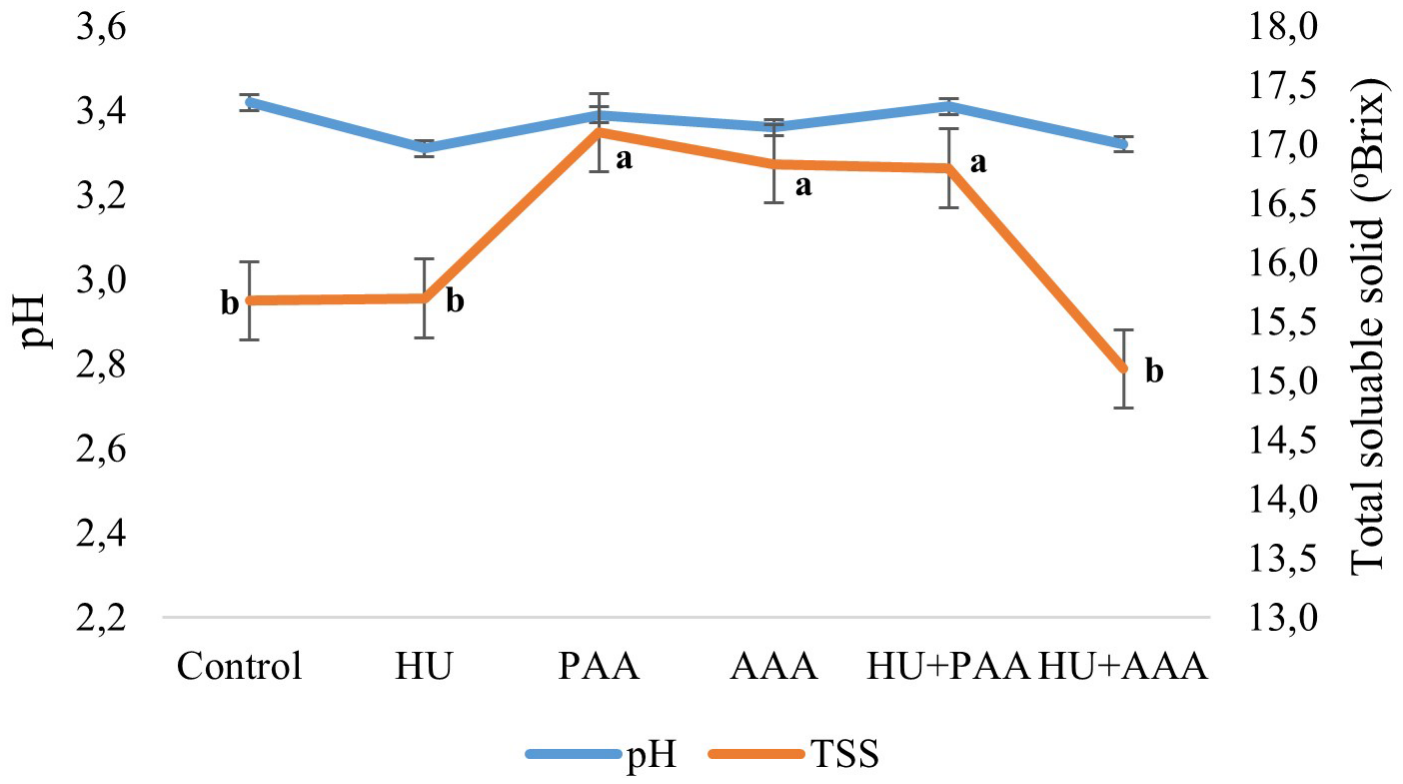
pH and total soluble solid content (brix,%) values are presented as the mean ± standard error bars. Different letters indicate significant differences (p ≤ 0.05) among different biostimulant applications.

**Figure 5:**
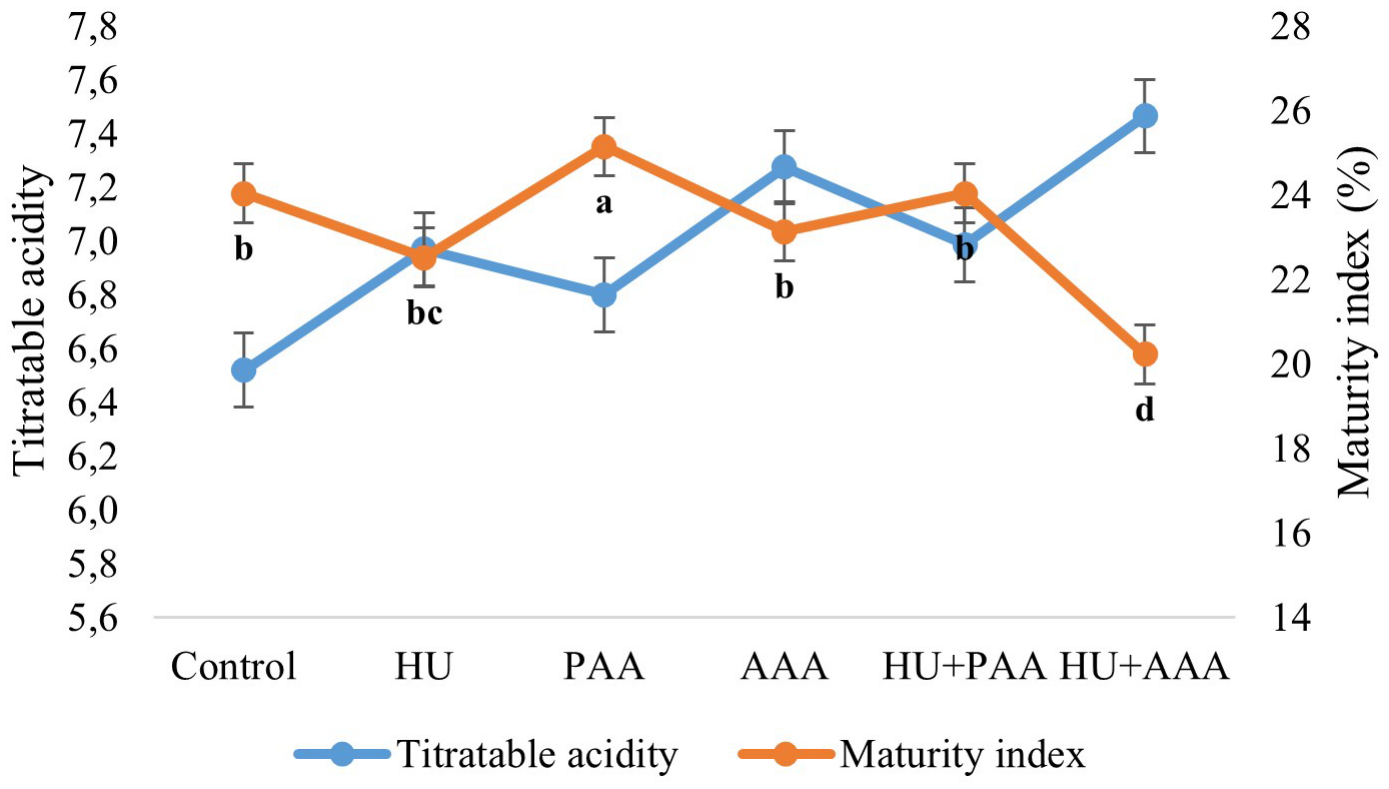
Titratable acidity (g L^-1^) and maturity index (%) values are presented as the mean ± standard error bars. Different letters indicate significant differences (p ≤ 0.05) among different biostimulant applications.

**Figure 6:**
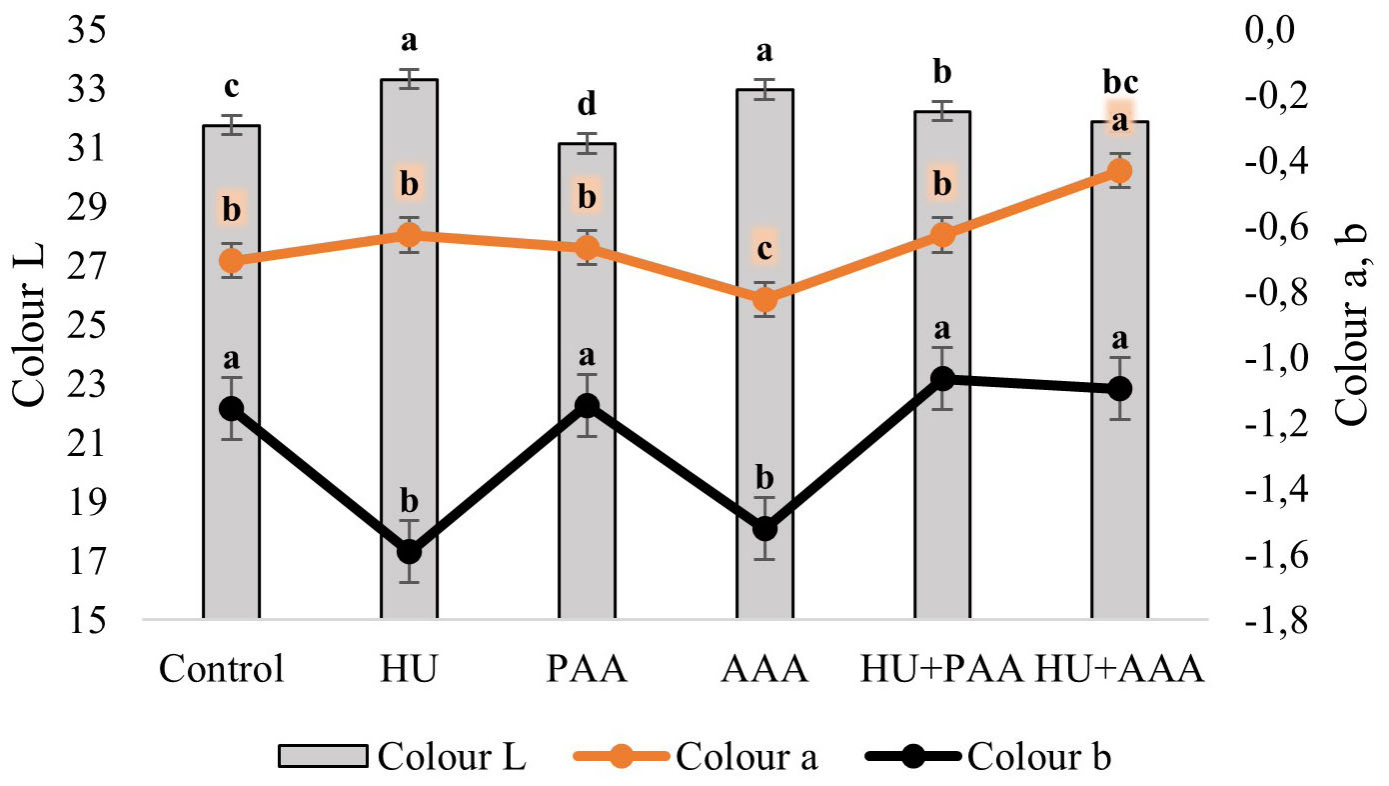
Colour parameters (L*, a* and b*) are presented as the mean ± standard error bars. Different letters indicate significant differences (p ≤ 0.05) among different biostimulant applications.

**Figure 7:**
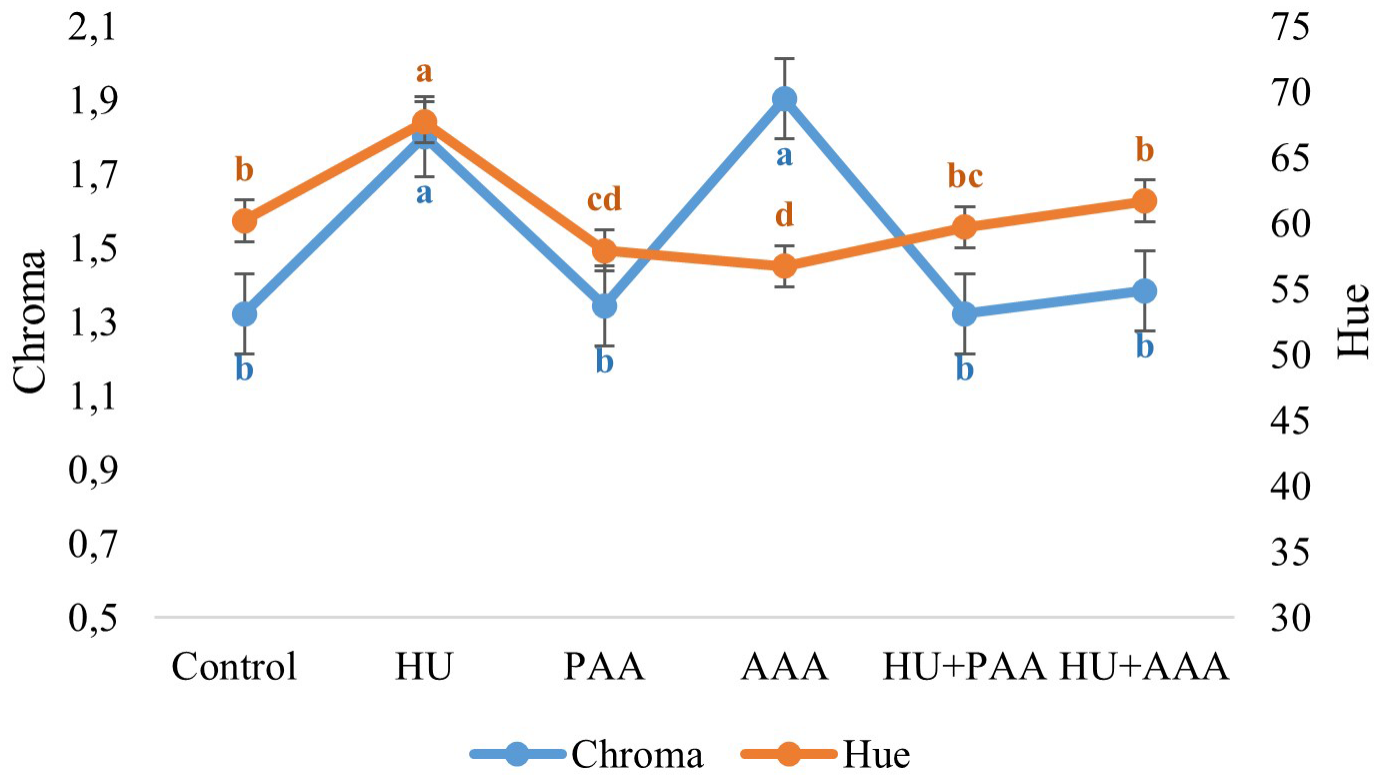
Chroma and hue values are presented as the mean ± standard error bars. Different letters indicate significant differences (p ≤ 0.05) among different biostimulant applications.

In this study, TSS, pH, TA and color parametres were also examined as quality criteria. Biostimulant applications had a statistically significant effect on all quality characteristics except pH and acidity. The pH values in the study were not affected by biostimulant applications and ranged between 3.26 and 3.37. In terms of total soluble solid the control, humic acid, and HU+PAA had lower values (15.68%, 15.70%, and 15.10%, respectively), while the PAA, AAA, and HU+PAA had higher values (17.10%, 16.83%, and 16.80%, respectively) (**Figure 4**). Titratable acidity values ranged from 6.52 to 7.42 depending on the biostimulant applications, and no statistically significant differences were detected between them. The lowest value for the maturity index was found in the HU+AAA (20.33%), while the highest value was found in the plant-derived amino acid (25.15%) (**Figure 5**). Significant differences were found between biostimulant applications in terms of berry colour values. The highest L value (lightness) was measured in humic acid (33.31%) and animal-derived amino acid (32.96%), while the lowest was measured in plant-derived amino acid (31.14%). The highest colour a* (redness) value was determined in the HU+AAA (-0.43), and the lowest value was in the animal-derived amino acid (-0.88). In terms of berry colour b* (yellowness) value, two distinct statistical groups were formed, with high values in the control, PAA, HU+PAA, and HU+AAA (respectively -1.16, -1.15, -1.07, -1.10), while low values were obtained in the humic acid and animal-derived amino acid (-1.59 and -1.52) (**Figure 6**). Animal-derived amino acid and humic acid had high Chroma values (1.90% and 1.80%), while the control, plant amino acid, HU+PAA, and HU+AAA had lower values (1.32, 1.34, 1.32, and 1.38, respectively) (**Figure 7**). Significant differences were also observed among the treatments in terms of hue angle (h^0^), with the highest hue value recorded in the humic acid (67.73) and the lowest value in the animal-derived amino acid (56.75) (**Figure 7**).

### Antioxidant components

**Figure 8:**
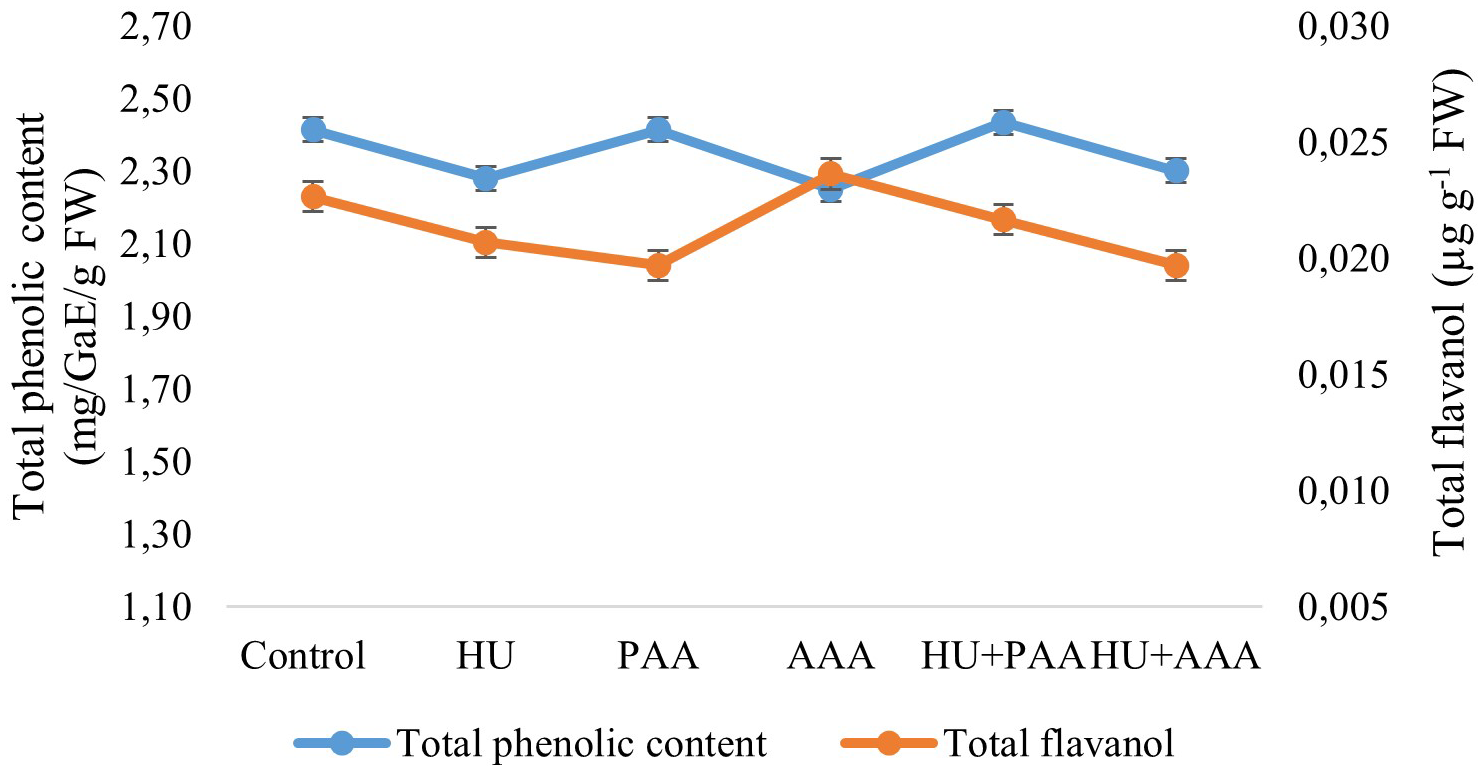
Total pheneolic content (mg/GaE/g FW) and total flavanol content (µg g^-1^ FW) values are presented as the mean ± standard error bars.

**Figure 9:**
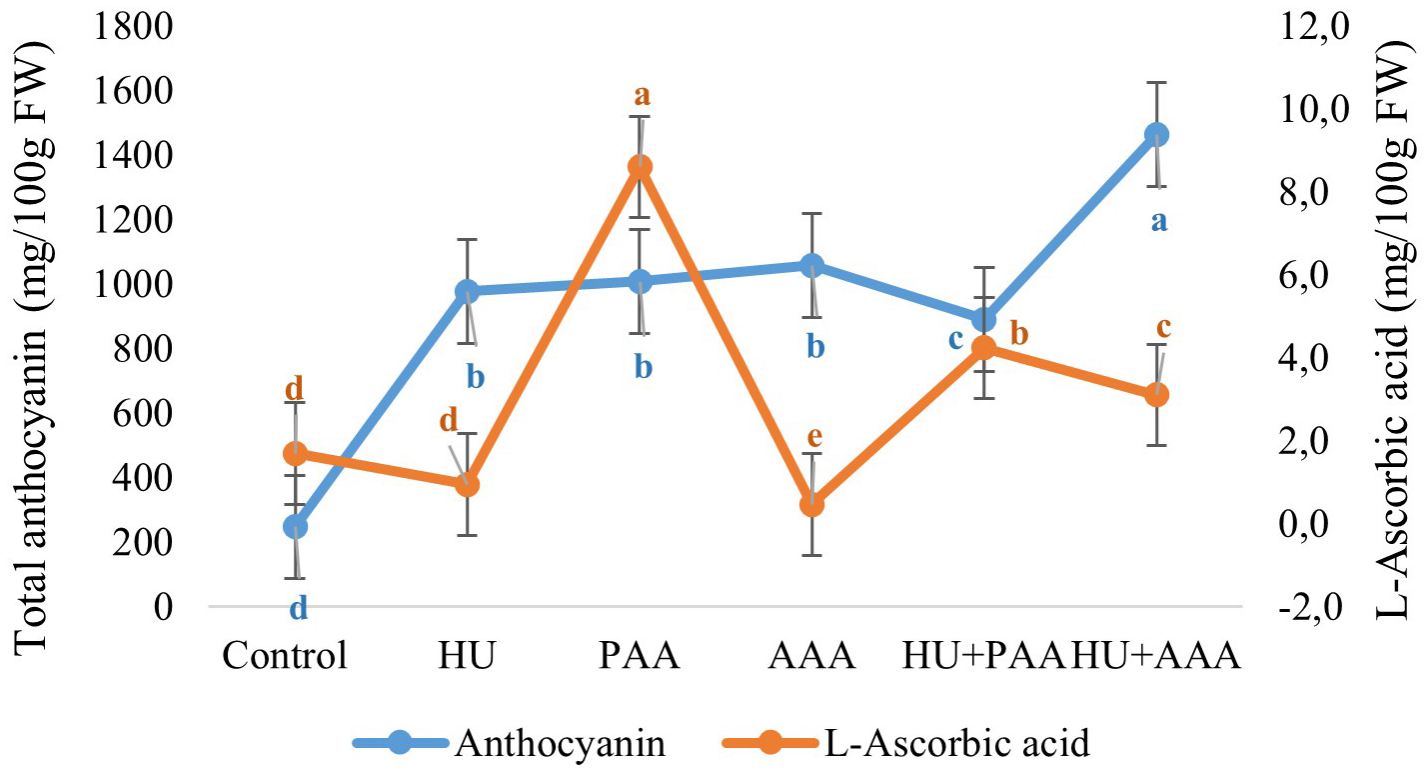
Total anthocyanin (mg/100g FW) and L-Ascorbic acid (mg/100g FW) values are presented as the mean ± standard error bars. Different letters indicate significant differences (p ≤ 0.05) among different biostimulant applications.

The analyses conducted on grapes revealed that the total phenolic compound content ranged between 2.25 and 2.43 mg GAE·g^-1^ FW, while the total flavanol content varied between 0.0197 and 0.0236 µg·CE g^-1^ FW. No statistically significant differences were observed among the biostimulant applications (**Figure 8**). Different biostimulant applications significantly affected the total anthocyanin content and L-ascorbic acid content. There were very large increases in total anthocyanin content with biostimulant applications. The value of 247 mg·100·g^-1^ FW in the control, HU+PAA was 891 mg·100·g^-1^ FW, humic acid was 977 mg·100·g^-1^ FW, plant-derived amino acid was 1008 mg·100·g^-1^ FW, and animal-derived amino acid was 1058 mg·100·g^-^ FW. The highest total anthocyanin content was determined to be 1463 mg·100·g^-1^ FW in the HU+AAA (**Figure 9**).

The highest L-ascorbic acid value was measured in the plant-based amino acid application (8.60 mg·100·g^-1^ FW), while the lowest value was measured in the animal-based amino acid application (0.47 mg·100·g^-1^ FW). HU+PAA (4.24 mg·100·g^-1^ FW) and HU+AAA (3.10 mg·100·g^-1^ FW) applications also increased the ascorbic acid content (**Figure 9**).

### Heat map analysis

**Figure 10:**
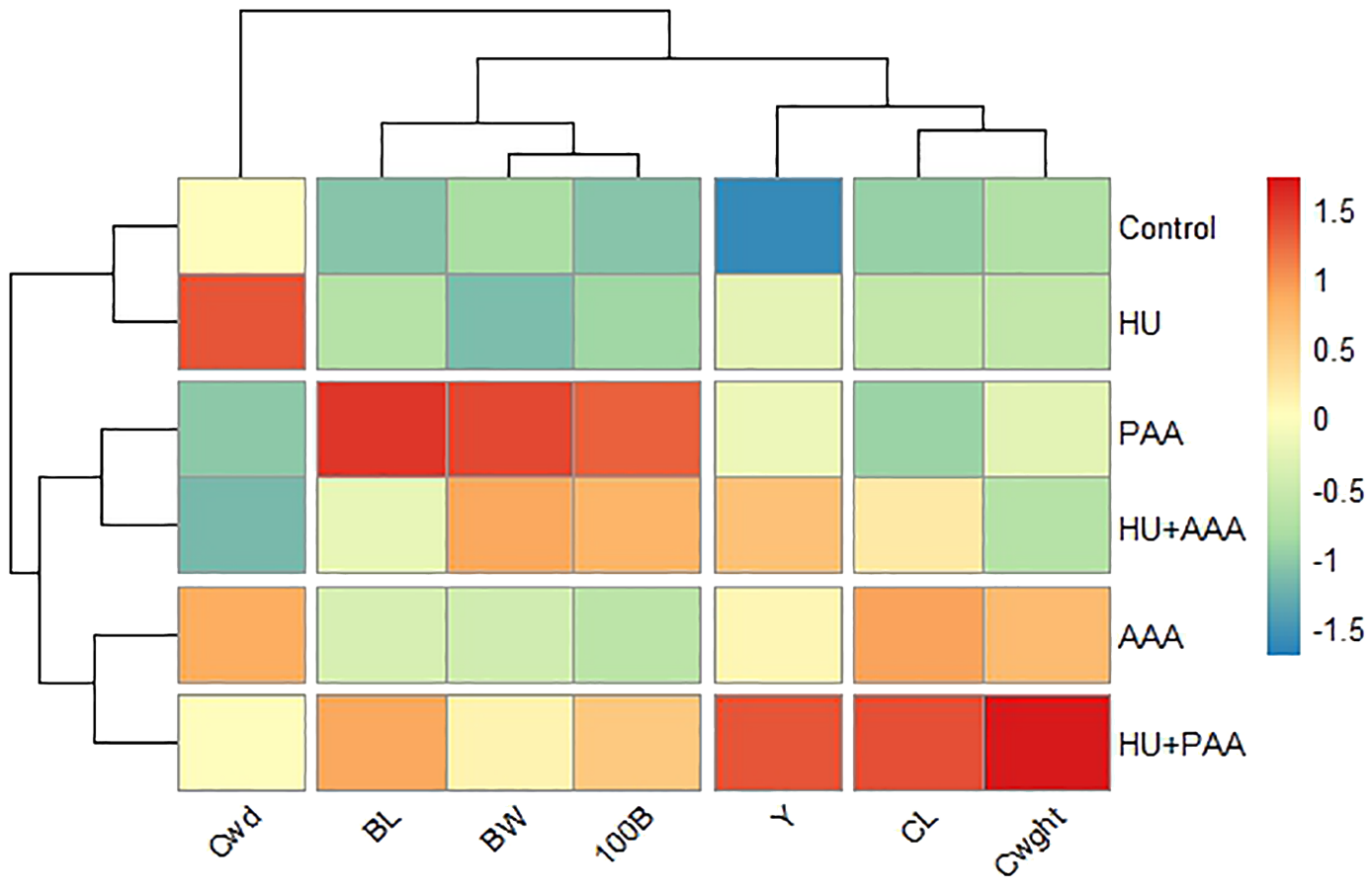
Heat map showing the effects of biostimulants tratments on yield parameters. BW, berry width; BL, berry length; Cwd, cluster width; CL, cluster length; Cwght, cluster weight; 100B, 100 berry weight and Y, yield.

The application of the HU+PAA resulted in favorable outcomes, as evidenced by the heat map, which was created based on yield parameters. The analysis revealed that the HU+PAA application exerted a positive influence on the fundamental yield components, including cluster length, cluster weight, and yield. The application achieved the highest Z-scores (**Figure 10**). Conversely, the PAA exhibited favorable outcomes in terms of berry length, 100-berry weight, and berry width. In contrast, the HU and AAA remained close to or below the average in the majority of parameters. In particular, it was found that all yield components were present at low levels in the control group. This finding indicates that biostimulants, particularly when employed in conjunction, are efficacious in promoting both growth and yield.

**Figure 11:**
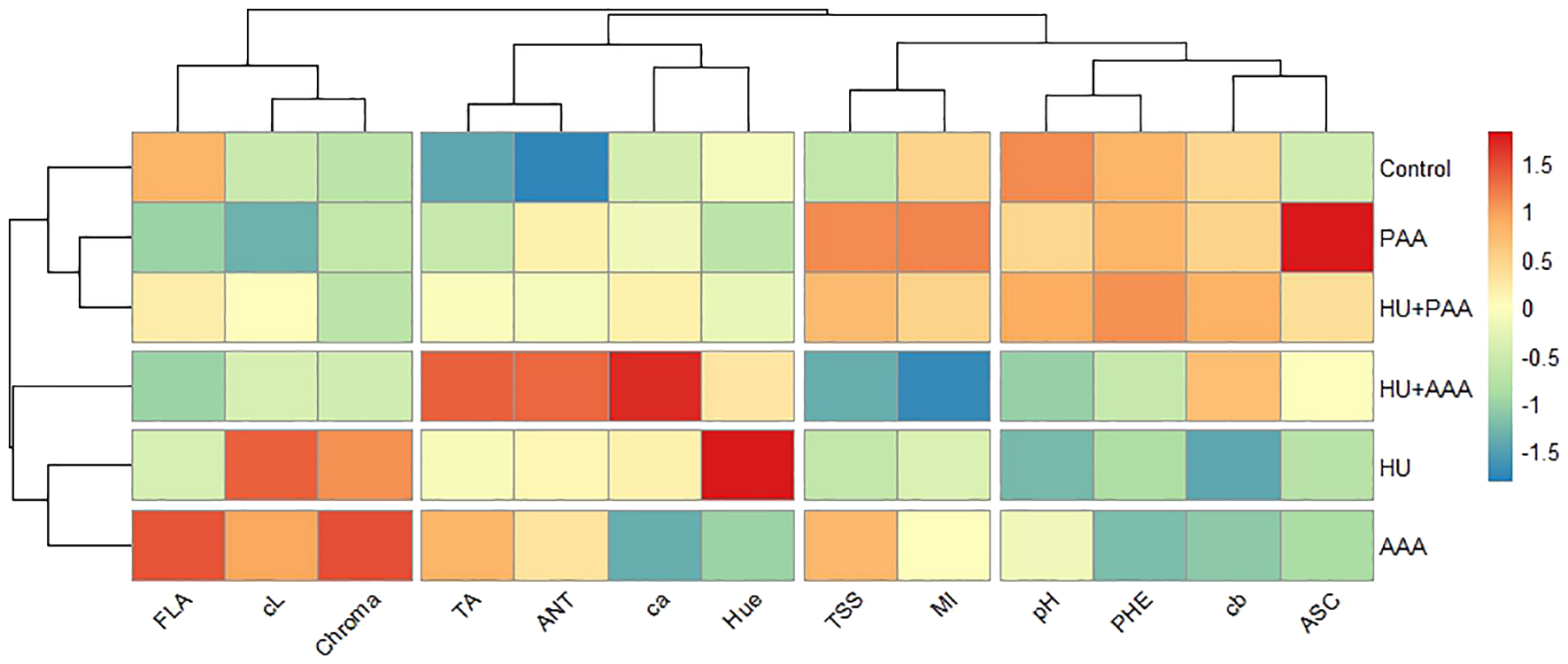
Heat map showing the effects of biostimulants tratments on quality parameters. TA, titratable acidity; FLA, total flavanol; ASC, L-Ascorbic acid; cL, berry color L; PHE, total phenolic matter; ANT, total anthocyanin; ca, berry color a; cb, berry color b; pH, pH değeri; TSS, total soluble solids; MI, maturity index.

Furthermore, a heat map was created to evaluate the relationships between quality parameters (**Figure 11**). The graph, which has been obtained through the process of normalization based on Z-scores, demonstrates that the HU+AAA exerted a positive effect on parameters related to berry pigmentation and juice balance, including anthocyanin, titratable acidity, and hue angle. The application of PAA was found to have a positive effect on characteristics related to antioxidant capacity and visual quality, such as phenolic compounds, ascorbic acid, pH, and skin color. The HU+PAA treatment plays a balancing role in many parameters, while the control and AAA demonstrated low performance in the majority of quality characteristics. The results obtained demonstrate the efficacy of biostimulants in both the processes of antioxidant and pigment production, thus supporting the hypothesis of synergistic effects arising from combination applications.

### Principal component analysis

**Figure 12:**
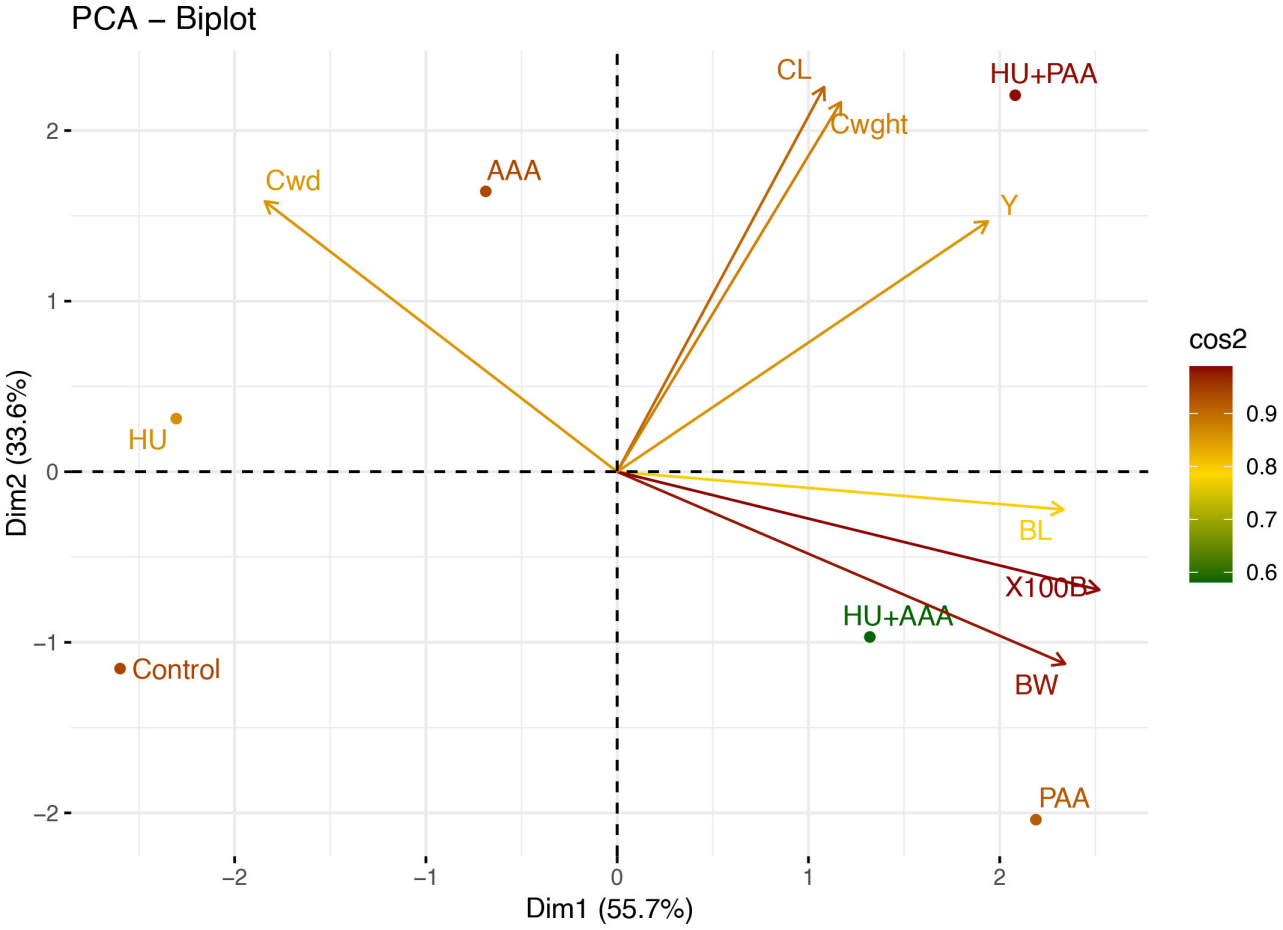
PCA (Principal Component Analysis) biplot graph showing the relationship between biostimulants treatments and yield parameters. BW, berry width; BL, berry length; Cwd, cluster width; CL, cluster length; Cwght, cluster weight; 100B, 100 berry weight and Y, yield.

PCA biplot analysis revealed that 89.3% of the variation in yield and morphological characteristics was concentrated in the first two components (Dim1: 55.7%, Dim2: 33.6%). The position of the HU+PAA along the yield, cluster length, and cluster weight vectors on the graph indicates a direct relationship between the applications and increased yield. The HU+AAA demonstrated a moderate effect, while the HU and AAA were located on the upper left axis, indicating a weaker effect. The plant amino acid (PAA) treatment demonstrated a closer relationship with parameters related to berry size, such as berry length, berry weight, and 100 berry weight. The control group was located far from all other treatments and was found to be negatively correlated with almost all yield components. The findings of this analysis provide statistical substantiation for the directional effects of the applications on the traits and the sources of variation (**Figure 12**).

**Figure 13:**
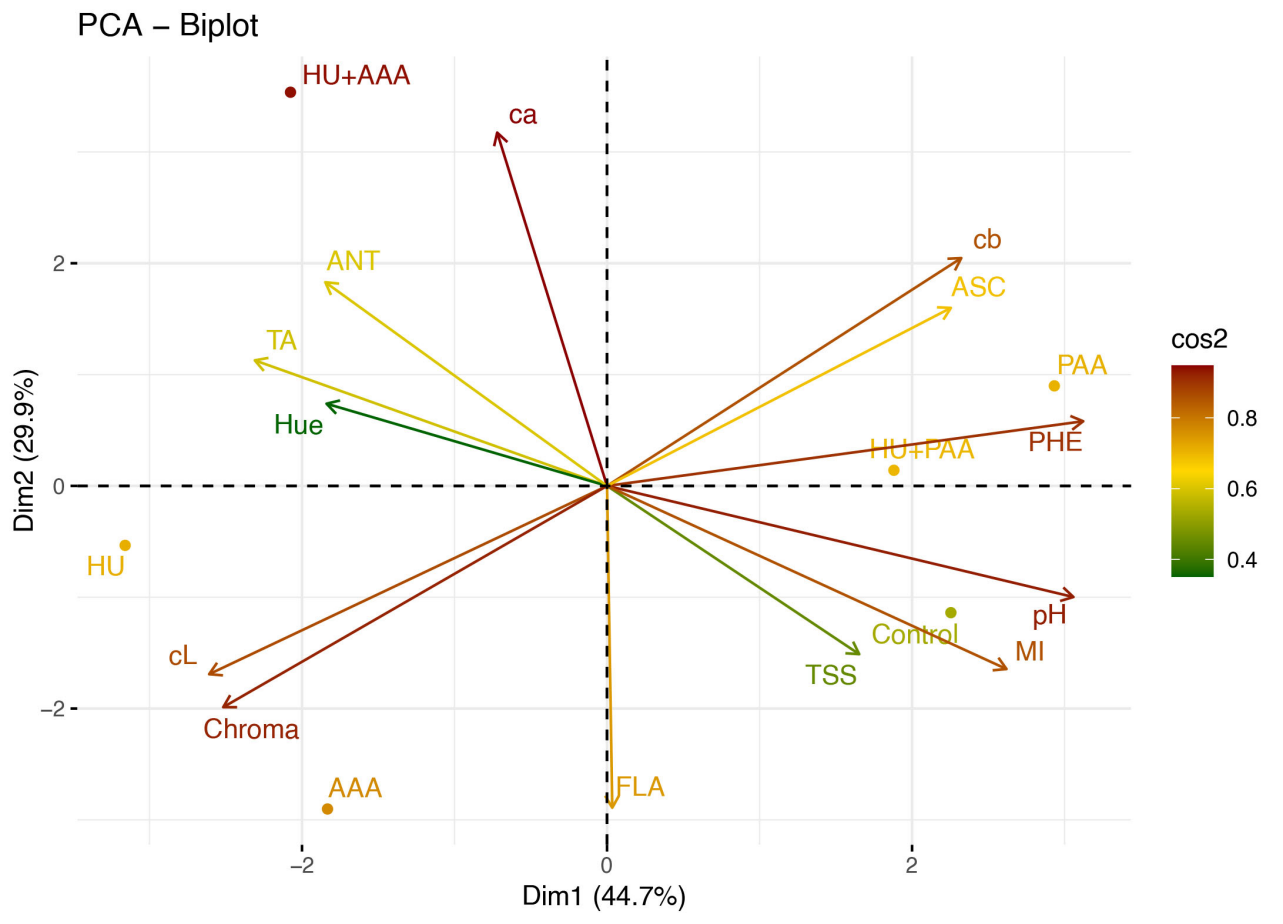
PCA (Principal Component Analysis) biplot graph showing the relationship between biostimulants treatments and quality parameters. TA, titratable acidity; FLA, total flavanol; ASC, L-Ascorbic acid; cL, berry color L; PHE, total phenolic matter; ANT, total anthocyanin; ca, berry color a; cb, berry color b; pH, pH değeri; TSS, total soluble solids; MI, maturity index.

The variation structure between the quality traits was evaluated in detail using principal component analysis (PCA), and two components (Dim1: 44.7%, Dim2: 29.9%) represented 74.6% of the data. In the PCA biplot graph, the HU+AAA treatment was positioned in close proximity to the anthocyanin, titratable acidity, and peel color parameters, thereby indicating a strong relationship with these characteristics. Conversely, the PAA and HU+PAA were positioned particularly in the direction of total phenolic, ascorbic acid, and pH, reflecting their positive effects on the biochemical contents determining quality. Conversely, the HU and AAA exhibited reduced effect levels, demonstrating minimal associations with color and flavonoid components such as total flavanol, chroma, and L (berry color). The results of the PCA demonstrate that combination applications provide a more balanced and multifaceted development in quality parameters than single applications (**Figure 13**).

## Discussion

Statistically significant differences were observed between the applications in terms of yield, with the highest value obtained with the application of humic acid and plant-derived amino acids. The effects of humic acid applications on yield and quality are based on the stimulation of various physiological and biochemical processes in plants. In particular, it is known that humic acids promote root development, thereby increasing water and nutrient uptake, which in turn leads to increased photosynthetic activity and, consequently, biomass growth (Canellas et al., 2011). Additionally, humic substances enhance the plant’s nutrient uptake efficiency by regulating cell membrane permeability and ion transport. Indeed, Popescu and Popescu (2018) reported, similarly to our study, that humic acid at doses of 30, 40, and 50 ml·L^−1^ caused an increase in the rapid growth of root cells, yield, TSS, and ATPase synthesis and activity. Similarly, Sabır et al. (2021) also reported that the application of humic acid increased yield in the Alphonse Lavallée grape variety and positively affected the marketability of table grapes.

In addition to humic acids, amino acids play important roles in plant physiology by directly participating in metabolic processes. It is known that plant-derived amino acids promote chlorophyll synthesis and increase photosynthetic capacity. In addition, amino acids increase plant resilience by supporting osmoregulation under stress conditions (Bulgari et al., 2019). It is also thought that amino acids accelerate protein synthesis, triggering cell division and expansion, thereby increasing berry size and cluster weight. Boselli et al. (2019) observed that both plant and animal protein hydrolysates have the capacity to enhance yield by over 20%. This study also noted the yield-enhancing effect of amino acid supplementation.

An analysis of the cluster weight and size revealed that the application of humic acid and plant derived amino acids exhibited a significant impact on the characteristics of the clusters. Specifically, the clusters treated with this combination demonstrated a length of 20.32 cm and a weight of 505.5 grams, which are noteworthy observations. As posited by Mohamed and Gouda (2016), the application of humic acid had a positive effect on cluster length and weight in the Flame Seedless variety. Similarly, as reported by Akın and Mostafa (2017), the application of humic acid was found to be effective in promoting cluster development in the Italia variety. Furthermore, Pepe (2019) observed that the administration of humic acid at a dose of 667 mL·5L to the Hafızali variety resulted in a remarkably elevated cluster weight of 743.9 g. The extant literature corroborates the observations on cluster development made in the present study. Boselli et al. (2019) reported that improvements in vine performance, including yield, cluster weight and number per vine, were dependent on the origin or application rate of protein hydrolysates. It can be said that the increase in yield resulting from the use of plant-derived amino acids is due to the organic nitrogen content of protein hydrolysates. It has been reported that small peptides in protein hydrolysates play a role in both hormone-like activity and the regulation of the nitrogen pathway (Ertani et al., 2009).

Examining the data in terms of berry size, the application of plant-derived amino acids produced the best results in terms of berry length and 100-berry weight. Sharma et al. (2023) and Colla et al. (2015) have stated that PAAs support plant growth, thereby increasing berry size and biomass. Our results on crop performance were similar to the positive effect of *Ascophyllum nodosum* seaweed extract extracts on ‘Thompson seedless’ grapes, which increased berry number, size and weight per bunch compared to the control *Ascophyllum nodosum* seaweed extract. increased berry size by 13% and berry weight by up to 39% (Norrie and Keathley, 2006). Belal (2015) demonstrated that humic acid positively influenced yield-determining factors such as cluster weight and length in the ‘King Ruby’ variety. Furthermore, Khan et al. (2012) found that a mixture of amino acids and seaweed increased the size of ‘Perlette’ grapes. In this context, the positive effects of applying plant amino acids on berry characteristics are particularly noteworthy in our study.

When yield values are evaluated in general in this study, it is seen that humic acid and plant-derived amino acids stand out in the yield components. This result can be attributed to the bioactivity of the applied compounds, amino acids and small peptides that can exhibit hormone-like activity and increase nitrogen and water uptake, thereby enhancing plant biomass productivity, and humic acid, which acts like a hormone (Colla et al., 2014; Popescu and Popescu, 2018).

Besides production parameters, biostimulants applications influenced fruit composition properties. Regarding the Brix value of total soluble solid (TSS), the highest values quality criteria were obtained with plant-derived amino acid and animal-derived amino acid applications. Boselli et al. (2019) reported that plant-derived amino acids from lupin (1.6  g L^−1^) protein hydrolysate increased the Brix value of TSS. Sharma et al. (2023) observed that biostimulant applications enhanced fruit quality, raising TSS values to 21.98 Brix. Furthermore, Ferrara and Brunetti (2010) stated that humic acid applications on soil improved quality parameters such as TSS and acidity. In our study, the stimulating effect of plant -derived amino acids on sugar accumulation was clearly observed.

The highest maturity index value was detected in the control group. This suggests that the application of humic acid and amino acids may have reduced the ripening rate due to their acidity-increasing effects. Titratable acidity data showed significant increases, particularly with animal-derived amino acid and humic acid+plant-derived animal acid applications. Frioni et al. (2018) reported that biostimulants have no significant effect on acidity or the TSS/acidity ratio but balance fruit quality. Bununla birlikte, Ferrara and Brunetti (2010) noted that humic acid application on soil leads to slight increase of Brix and the statistically significant reduction of titratable acidity.

In terms of color parameters reflecting berry color and pigmentation, the highest Chroma value was recorded with animal-derived amino acid applications and humic acid applications. These applications were found to provide a more vibrant berry color. McGuire (1992) observed that chroma is directly related to color saturation, and Mutlu and Ergüneş (2008) emphasized that this value is crucial for marketability. Additionally, it was observed that humic acid application shifted the color tone towards the blue-green spectrum in terms of hue angle.

Phenolic compounds, a group of secondary metabolites, have various biological effects, including antioxidant and antibacterial activities, and play an important role in neutralising free radicals and suppressing oxygen molecules through the displacement or decomposition of peroxides (Baydar et al., 2011). Additionally, anthocyanins provide protection against oxidative stress in plants; they scavenge ROS to reduce oxidative damage through their aromatic ring, bound free OH groups, and chelating metals (Salama et al., 2023). In our study, the highest total anthocyanin content (1463 mg·100·g^-1^ FW) was detected in the humic acid animal-derived amino acid combination. The anthocyanin content increased 5.9 times compared to the control and had a darker skin colour. This finding demonstrates the synergistic effect of combinations of humic acid and amino acids. The increase in the accumulation of these compounds due to the combination of humic acid and animal amino acids can be attributed to the promotion of the expression of key enzymes such as phenylalanine ammonia lyase (PAL). As a result, colour development in the fruit skin becomes more pronounced and marketability increases. Similarly, Deng et al. (2019) demonstrated that the application of the biostimulant Sunred (Biolchim Co., Italy) before veraison in “Red Globe” grapes resulted in a 1.4-fold increase in total anthocyanin content compared to the control. Boselli et al. (2019) reported that amino acid applications can increase total anthocyanin content by up to 200%. Sharma et al. (2023) emphasized that biostimulants positively affect phenolic compounds and antioxidant capacity, and combination applications further enhance this effect. Similarly, Khan et al. (2012) reported that multiple amino acid applications increased anthocyanin levels in the Perlette variety. The increase in anthocyanins is generally associated with the activation of the phenylpropanoid pathway under stress stimulation. The accumulation of these compounds due to the combination of humic acid and animal amino acids may be linked to the promotion of the expression of key enzymes such as phenylalanine ammonia lyase (PAL). As a result, colour development in the fruit peel becomes more pronounced and marketability increases.

While no statistical differences were observed in total phenolic content or flavanol quantities, the HU+PAA application stood out for total phenolic content, and animal-derived amino acids were notable for flavanol quantity. Taken together with Ojeda et al. (2002) study indicating that water stress can increase phenolic compound accumulation, these results suggest that application timing and environmental conditions also influence phenolic compounds. Frioni et al. (2019) reported that biostimulants improve fruit quality and extend shelf life by promoting phenolic compound synthesis.

The highest L-ascorbic acid content was obtained with the highest level of plant-derived amino acid application. Ascorbic acid is a powerful antioxidant in plants and plays an important role in stress tolerance (Sharma and Dietz, 2006). Bulgari et al. (2019) observed that amino acids promote ascorbic acid synthesis in plants and positively impact quality parameters. Van Oosten et al. (2017) reported that biostimulants enhance ascorbic acid accumulation under abiotic stress conditions, thereby supporting plant resistance. In particular, the contribution of plant-based amino acids to high ascorbic acid content is related to the antioxidant role of this compound. Ascorbic acid protects cell membranes and enzyme systems by neutralising reactive oxygen species (ROS), thereby contributing positively to fruit quality and shelf life (Sharma and Dietz, 2006). In addition, ascorbic acid can support the pigmentation process by acting as a cofactor in anthocyanin biosynthesis.

Heat map analyses visually demonstrate the responses of yield and quality traits to biostimulants. The application of HU+AAA was found to significantly increase parameters such as anthocyanin, colour a, hue, and titratable acidity, while PAA application was positively correlated with pH, phenolic compounds, and ascorbic acid. These results are consistent with those of Ferrara and Brunetti (2010), who reported that humic acid enhances anthocyanin synthesis. Sharma et al. (2023) emphasized that heat map analyses are an effective tool for visualizing the effects of biostimulants on yield and quality parameters, particularly for revealing the synergistic effects of combination applications.

PCA analyses clearly distinguished the effects of biostimulant applications on quality and yield parameters. Notably, the humic acid+plant-derived amino acid combination showed a strong correlation with quality parameters such as phenolic content, ascorbic acid, and pH, as well as yield parameters such as cluster weight, cluster length, and yield amount. This clearly demonstrates the synergistic effects of combination applications. Frioni et al. (2019) noted that PCA analyses are an important tool for understanding the mechanisms of action of biostimulants, particularly in revealing relationships between multiple parameters. Similarly, Sharma et al. (2023) emphasized that such biostimulant combinations activate physiological processes more strongly.

As a result, it has been observed that these applications stimulate multifaceted physiological and biochemical processes in plants with bioactive components from different sources, thereby increasing quality and yield components. This effect is particularly pronounced in combination applications (humic acid + plant-animal derived amino acids), demonstrating a synergistic effect. The application of biostimulants to vines to avoid certain abiotic stresses and improve grape and wine quality has become one of the most widely used practices among wine and table grape producers in many wine-growing regions around the world in recent years. In our study, two-year averages show that the application of biostimulants has positive effects on yield, cluster and berry characteristics, berry color, and biochemical quality parameters in the Öküzgözü wine grape variety. However, it should be noted that this effect varies depending on factors such as the source of the biostimulant, application timing, and dosage (Lisiecka et al., 2011; Popescu and Popescu, 2018). Van Oosten et al. (2017) noted that biostimulant effectiveness depends on environmental conditions and application strategies and that optimized protocols need to be developed. The obtained data indicate that biostimulants can successfully replace chemical fertilizers in sustainable viticulture.

## Data Availability

The original contributions presented in the study are included in the article/supplementary material. Further inquiries can be directed to the corresponding author.
